# Transcriptional profile and Epstein-Barr virus infection status of laser-cut immune infiltrates from the brain of patients with progressive multiple sclerosis

**DOI:** 10.1186/s12974-017-1049-5

**Published:** 2018-01-16

**Authors:** Caterina Veroni, Barbara Serafini, Barbara Rosicarelli, Corrado Fagnani, Francesca Aloisi

**Affiliations:** 1 Department of Neuroscience, Istituto Superiore di Sanità, Viale Regina Elena 299, 00161 Rome, Italy; 2 Centre for Behavioural Sciences and Mental Health, Istituto Superiore di Sanità, Viale Regina Elena 299, 00161 Rome, Italy

**Keywords:** Multiple sclerosis, Innate immunity, Adaptive immunity, Epstein-Barr virus, Laser capture microdissection, Gene expression, Multivariate analysis

## Abstract

**Background:**

It is debated whether multiple sclerosis (MS) might result from an immunopathological response toward an active Epstein-Barr virus (EBV) infection brought into the central nervous system (CNS) by immigrating B cells. Based on this model, a relationship should exist between the local immune milieu and EBV infection status in the MS brain. To test this hypothesis, we analyzed expression of viral and cellular genes in brain-infiltrating immune cells.

**Methods:**

Twenty-three postmortem snap-frozen brain tissue blocks from 11 patients with progressive MS were selected based on good RNA quality and prominent immune cell infiltration. White matter perivascular and intrameningeal immune infiltrates, including B cell follicle-like structures, were isolated from brain sections using laser capture microdissection. Enhanced PCR-based methods were used to investigate expression of 75 immune-related genes and 6 EBV genes associated with latent and lytic infection. Data were analyzed using univariate and multivariate statistical methods.

**Results:**

Genes related to T cell activation, cytotoxic cell-mediated (or type 1) immunity, B cell growth and differentiation, pathogen recognition, myeloid cell function, type I interferon pathway activation, and leukocyte recruitment were found expressed at different levels in most or all MS brain immune infiltrates. EBV genes were detected in brain samples from 9 of 11 MS patients with expression patterns suggestive of in situ activation of latent infection and, less frequently, entry into the lytic cycle. Comparison of data obtained in meningeal and white matter infiltrates revealed higher expression of genes related to interferonγ production, B cell differentiation, cell proliferation, lipid antigen presentation, and T cell and myeloid cell recruitment, as well as more widespread EBV infection in the meningeal samples. Multivariate analysis grouped genes expressed in meningeal and white matter immune infiltrates into artificial factors that were characterized primarily by genes involved in type 1 immunity effector mechanisms and type I interferon pathway activation.

**Conclusion:**

These results confirm profound in situ EBV deregulation and suggest orchestration of local antiviral function in the MS brain, lending support to a model of MS pathogenesis that involves EBV as possible antigenic stimulus of the persistent immune response in the central nervous system.

**Electronic supplementary material:**

The online version of this article (10.1186/s12974-017-1049-5) contains supplementary material, which is available to authorized users.

## Background

Multiple sclerosis (MS) is a chronic inflammatory disease of the central nervous system (CNS) resulting from a complex interaction between genetic, lifestyle, and environmental risk factors, the latter including infectious and non-infectious factors [1]. Influx of leukocytes into the CNS and activation of the CNS innate immune system are the key pathogenic processes leading to demyelination, neurodegeneration, and gliosis in MS [2–5]. Blood-derived immune cells accumulate mainly in CNS connectival spaces [i.e., the perivascular space of post-capillary venules in the white matter (WM) and, less frequently, gray matter (GM) and the subarachnoid space lined by the leptomeninges] or extravasate in the CNS parenchyma, as shown by analysis of postmortem brain samples and, in a few cases, brain biopsies from MS patients [2–6]. The immune infiltrate of the MS brain is dominated by lymphocytes, mainly T cells and variable numbers of B cells and plasma cells, and by myeloid cells, for the most part macrophages [2–5]. In the subarachnoid space, chronic inflammation leads to formation of lymphoid-like structures resembling B cell follicles [6, 7]. Innate immune cells, like dendritic cells (DC), myeloid DC, and plasmacytoid DC (pDC), and natural killer (NK) cells are a minor, variable component of the CNS immune cell infiltrate [8–12].

The innate and adaptive immune systems converge into three major kinds of cell-mediated effector immunity that have been categorized as type 1, type 2, and type 3 based on expression of transcription factors and cytokine production (reviewed in [13]). Type 1 immunity consists of T box expressed in T cells (Tbet)+/Eomesodermin (EOMES)+ interferon (IFN) γ producing CD4+ T helper 1 (Th1) cells, CD8+ cytotoxic T cells (Tc1), and group 1 innate lymphoid cells (ILCs) (ILC1s and NK cells) that protect against intracellular microbes through direct killing of infected cells and activation of macrophages. Type 2 immunity consists of GATA-3+ CD4+ Th2 cells, CD8+ Tc2 cells, and ILC2s that produce interleukin (IL)-4, IL-5, and IL-13 and induce mast cell, basophil, and eosinophil activation, as well as IgE antibody production, protecting against helminth parasites. Type 3 immunity is mediated by retinoic acid-related orphan receptor (ROR) γt+ CD4+ Th17 cells, CD8+ Tc17 cells, and ILC3s producing IL-17, IL-22, or both, which activate innate and resident tissue cells and recruit neutrophils, providing protection against extracellular bacterial and fungal infections. Type 1 and type 3 immunity have been implicated in autoimmune diseases, whereas type 2 responses mediate allergic diseases [13].

MS has been traditionally considered a Th1-mediated disease [14], and IFNγ is a major pro-inflammatory cytokine produced in the MS brain [5, 15–17]. However, it is also known that the CNS immune infiltrate is dominated by CD8+ T cells displaying signs of local activation, like clonal expansion and expression of cytolytic enzymes [2, 5, 18–22]. Both MS lesion analysis and experimental studies have highlighted a key role for CD8+ T cells in neurodegeneration [23, 24]. Recent studies point to a pathogenic role for granulocyte-macrophage colony-stimulating factor (GM-CSF) producing T cells in MS via enhanced myeloid cell recruitment and activation [25, 26], and GM-CSF producing CD4+ and CD8+ T cells have been identified in MS brain lesions [27]. Myeloid cells recruited to the CNS and the CNS resident microglia are implicated in MS pathogenesis as antigen-presenting cells, source of pro-inflammatory cytokines, and effectors of myelin destruction [11, 26, 28].The role of IL17- and IL17/IFNγ-producing CD4+ and CD8+ T cells in MS pathogenesis is debated, as conflicting data exist on the frequency of these cell subsets in the brain and cerebrospinal fluid [5, 17, 25, 29–32]. It has been suggested that Th17 cells may be implicated in the formation of ectopic lymphoid-like tissue in the inflamed CNS [32, 33]. Recently, clonally expanded CD4+ and CD8+ T cells with type 2  immunity functional features were identified in WM lesions characterized by complement and immunoglobulin deposition (pattern II brain lesions) [34].

The specific target of immune-mediated injury in MS remains undetermined. Autoimmunity is pursued as the main trigger of chronic CNS inflammation [35, 36], but other pathogenic mechanisms, including infections, are also being explored [37]. The ubiquitous herpesvirus Epstein-Barr virus (EBV) is the main environmental risk factor for MS [38], and immune reactivity toward EBV is higher in MS patients than in the healthy population [39, 40]. However, it is still unknown whether EBV itself or the immune response to EBV may facilitate, induce, or modulate the disease. Due to its ability to infect and promote the transformation of B cells, as well as to elicit potentially pathogenic immune responses, EBV infection may contribute to MS through different mechanisms. These include immortalization of autoantibody-producing B cell clones, molecular mimicry, and immunopathology, namely the persistent attempt of the immune system to get rid of an infection at the expenses of tissue integrity [40, 41]. While several groups have reported absence or paucity of EBV in postmortem MS brain samples [42–45], we have repeatedly shown not only presence of EBV-infected B-lineage cells but also EBV latency disruption and reactivation in the MS brain [15, 46–50]. Neither EBV RNA/protein nor deregulated EBV infection was detected in brain tissues from patients with other infectious and non-infectious neuroinflammatory diseases [15, 46], ruling out the possibility that an active EBV infection in the CNS is the general consequence of immune cell invasion and local activation.

If MS were the result of an immunopathological response aimed at killing CNS-infiltrating EBV-infected B cells*,* a relationship should exist between the local immune milieu and EBV status. Hence, we reasoned that gene expression profiling of CNS immune infiltrates should be a valid approach to investigate such a link. Here, we have combined immunohistochemistry, laser capture microdissection (LCM), and enhanced polymerase chain reaction (PCR)-based methods to study expression of a large number of selected cellular and EBV genes in well-characterized immune infiltrates isolated from postmortem brain sections of patients with progressive MS, mainly secondary progressive MS. The data obtained were analyzed using univariate and multivariate statistical methods to check for possible associations between gene expression, immune infiltrate localization or organization, and EBV status and to identify immune activation signatures.

## Methods

### Tissues and sample selection

Postmortem frozen tissue blocks (4 cm^3^ each) from the cerebral hemispheres of MS patients were obtained from the UK Multiple Sclerosis Tissue Bank at Imperial College London. Twenty-two cases who died in the progressive phase of MS, mainly during secondary progressive MS, and with postmortem delay ≤ 26 h were selected (Additional file 1). Use of postmortem human brain material was approved by the Ethics Committee of Istituto Superiore di Sanità. To check for RNA quality, RNA was extracted from a pool of four 20-μm sections (corresponding to 20–35 mg of tissue wet weight) cut from each tissue block using RNeasy mini kit (Qiagen, Valencia, CA). RNA integrity number (RIN) was evaluated using Agilent 2100 Bioanalyzer (Agilent Technologies, Santa Clara, CA). Brain samples that showed RIN values ≥ 6 were used for neuropathological analysis; among these, only samples containing prominent immune infiltrates in the WM and/or meninges were used for the subsequent LCM procedure (Additional file 1).

A snap-frozen human hilo-pulmonary lymph node was obtained from Dr. Egidio Stigliano (Department of Pathological Anatomy, Policlinico A. Gemelli, Rome) and used in preliminary experiments to assess the specificity and uniformity of the preamplification real-time RT-PCR method.

### Immunohistochemistry

Cryosections (10 μm) cut from each brain tissue block were stained with hematoxylin and eosin (H&E) to evaluate the degree of meningeal and WM immune cell infiltration and immunostained for myelin-oligodendrocyte glycoprotein (MOG) and major histocompatibility complex (MHC) class II molecules to assess the extent of demyelination and inflammatory activity, as previously described [6, 7, 47–50]. The WM areas from which immune infiltrates were isolated are the following: active WM lesions, characterized by the presence of macrophages/activated microglia throughout the lesion area; chronic active lesions with a hypocellular lesion center with macrophages/activated microglia confined to the lesion border; and WM areas containing immune infiltrates and activated microglia but otherwise normal or rarefied myelin (Additional file 2).

The cellular composition of immune infiltrates was investigated using immunohistochemistry and/or indirect immunofluorescence and monoclonal antibodies specific for CD20, CD3, CD8, CD35, CD68 (DakoCytomation, Glostrup, Denmark), and rabbit polyclonal antibodies against Ig A, M, G (DakoCytomation), and CD8 (Thermo Fisher Scientific, Rockford, USA), as described [6, 7, 15, 49]. B cell follicle-like structures in the subarachnoid space were characterized as B cell aggregates containing CD35+ stromal cells (Additional file 2) [6, 15]. Anti-CD20 monoclonal antibody and anti-CD8 polyclonal antibody were routinely used to stain sections before and after the series of sections used for LCM.

### Laser capture microdissection and RNA extraction

Eight to 10 serial brain sections (10 μm) were cut with a cryostat in RNAse-free conditions, mounted on membrane-coated microscopy nuclease and nucleic acid free slides (MMI AG, Glattbrugg, Switzerland), and subjected to rapid nuclear staining and dehydration procedures (Arcturus Histo Gene Staining Solution, Life Technologies, Grand Island, NY), according to the manufacturer instructions. Sections were air-dried for 1 h, and LCM was performed using a laser microdissector SL Cut (MMI AG) equipped with a UV-Cut SL Microtest software and a Nikon Eclipse TE2000-S microscope. The beam parameters were as follows: exposure time 1/250; focus 80%; energy 87%; laser speed 16%; objective ×20. The procedure was performed in RNase-free conditions. The immune infiltrates selected for LCM were perivascular cuffs with > 3 cell layers localized in active WM lesions, at the hypercellular border of chronic active WM lesions and in WM areas with rarefied or normal-appearing myelin and activated microglia; B cell follicle-like structures (containing several hundred up to a thousand cells in a section); and diffuse immune infiltrates (> 500 cells per mm of intact meninges) localized in the subarachnoid space lined by the leptomeninges. The same infiltrated area was isolated from 8 to 10 serial sections and the fragments collected in a single cap. The WM and GM parenchyma adjacent to the perivascular and meningeal immune infiltrates, respectively, were also collected from 1 to 10 serial sections (median number = 4). The pooled microdissected areas ranged from 38,000 to 1,545,000 μm^2^ (median value = 208,000 μm^2^) for meningeal infiltrates; from 45,000 to 817,800 μm^2^ (median value = 157,400 μm^2^) for WM perivascular infiltrates; and from 162,000 to 1,000,000 μm^2^ (median value = 660,000 μm^2^) for WM and GM areas. After microdissection, the pooled tissue fragments of each series were incubated immediately in 50 μl of RNA stabilizing, extraction buffer (PicoPure RNA isolation kit, Arcturus, Life Technologies) at 42 °C for 30 min and centrifuged at 800×*g* for 2 min. Lysates were stored at − 80 °C until use.

### Preamplification real-time reverse transcription PCR and droplet digital PCR

Total RNA was extracted from the microdissected samples using Picopure RNA isolation kit (Arcturus, Life Technologies) following the manufacturer’s instructions, including Qiagen DNase treatment, and immediately reverse transcribed using the High Capacity Reverse Transcription kit with RNase inhibitor (Life Technologies). cDNA was diluted to a final volume of 50 μl and split into four 12.5 μl aliquots. To increase the number of targeted copies, cDNA was preamplified for a total of 75 cellular and 6 viral transcripts (maximum of 22–26 transcripts/aliquot) using a pool of 100-fold diluted 20× Taqman Gene Expression Assays and the TaqMan PreAmp Master Mix (Life Technologies) at the following thermal conditions: 50 °C for 2 min and 95 °C for 10 min, followed by 95 °C for 15 s and 1 min at 60 °C for 14 cycles. The inventoried assays used for cellular gene expression analysis and the self-designed primer and probe sequences specific for viral transcripts are listed in Additional files 3 and 4, respectively. The final preamplification product was diluted 1:5 and used as template for downstream PCR analysis.

For the study of cellular genes, preamplified cDNA was analyzed in triplicate by real-time PCR (ABI PRISM 7500 Real-Time PCR System, Life Technologies) using Taqman Gene Expression Master Mix (Life Technologies) and the same TaqMan Gene Expression Assays used in the preamplification step. Gene expression levels are expressed as 2^−ΔCt^ value relative to the endogenous GAPDH mRNA. The specificity and uniformity of the preamplification reaction was verified independently for each target gene, as previously described [51], using cDNA from a non-pathological human lymph node. Briefly, preamplification uniformity values were calculated for each target gene as the difference (indicated as ΔΔCt) between non-preamplified ΔCt data and preamplified ΔCt data. A ΔΔCt = ± 1.5 was set as a quality threshold for an acceptable preamplification reaction, according to the TaqMan PreAmp Master Mix guide. All the investigated gene assays showed a preamplification uniformity value close to zero (mean ΔΔCt value ± SD = 0.19 ± 0.32), indicating optimal preamplification conditions. A no template control that omitted RNA but contained all the other essential components of the amplification reaction was included as negative control during the whole real-time PCR procedure to check for possible reagent and primer contamination.

The efficiency of the EBV self-designed gene expression assays was checked in a previous study [51]. Aiming to minimize background cellular signals and ensure optimal quantification of viral RNA, PreAmp-droplet digital (dd) PCR was used to evaluate EBV gene expression. Four microliters of preamplified cDNA (obtained as described above) were used as template and amplified in triplicate for six EBV transcripts (EBER1, EBNA3A, LMP1, LMP2A, BZLF1, gp350/220) and human GAPDH using Droplet PCR Supermix no dUTP (Bio-Rad) and the same self-designed Taqman assays used in the preamplification step at the following thermal cycling conditions: 10 min at 95 °C and 30 s at 94 °C followed by 1 min at 57 °C for 40 cycles and 10 min at 98 °C; a no template control was always included. Viral RNA was analyzed using Bio-Rad QX200 droplet digital PCR System, and EBV transcript levels were normalized to the internal control GAPDH. The specificity of the EBV self-designed gene expression assays was assessed in preliminary experiments using the EBV+ lymphoblastoid cell line L5 and the EBV-negative B-lymphoma cell line BJAB [51] (Additional file 5). The data obtained with PreAmp-ddPCR were also compared with those obtained with the previously validated PreAmp real-time PCR method [51]. One-hundred nanograms of cDNA from EBV+ and EBV− cells were preamplified for EBV and GAPDH transcripts (14 cycles) and then analyzed by real-time RT-PCR and ddPCR using ABI PRISM 7500 and Bio-Rad QX200 System, respectively. As shown in Additional file 6, the two techniques provide comparable quantitative data.

### Statistical analysis

Univariate and multivariate statistical methods were applied in a mixed way to address the study questions. Between-group comparisons for continuous variables were performed using Mann–Whitney and Kruskal–Wallis tests in their cluster-adjusted version to account for multiple (i.e., correlated) measures within cases. Comparisons between categorical variables were performed using Fisher’s exact probability test. Continuous and categorical variables were summarized as means and standard deviations or medians and interquartile ranges, and percentages, respectively. In order to search for underlying groupings of cases based on differentially expressed genes and to unravel coordinated gene expression patterns, data from meningeal and WM infiltrates were also examined from a multivariate perspective and were subjected to cluster and factor analysis. Given the relatively low number of observations available, it was deemed prudent to reduce the number of variables (i.e., genes) prior to multivariate modeling. In this respect, the following procedure was applied: (i) rarely expressed genes were excluded, (ii) only genes with moderate to high Spearman correlation with at least two other genes were entered in factor analysis (*n* = 41), and (iii) cluster analysis was performed using the same genes selected for factor analysis. Hierarchical cluster analysis was conducted using the average linkage method with the Euclidean distance measure to calculate clusters. In this analysis, mean within-case gene expression levels over multiple measures were considered. The optimal number of clusters was assessed based on the Calinski/Harabasz pseudo-*F* index and the Duda/Hart Je(2)/Je(1) index stopping rules. Dendrogram plots were used to display the clustering results. Exploratory factor analysis (EFA) was carried out through the principal factor extraction method with orthogonal varimax rotation. The decision regarding the number of factors to retain was guided by eigenvalues exceeding 1.0 and visual inspection of the scree plot. For the interpretation of the factor solution, only those original variables having factor loadings higher than 0.5 in absolute value  were considered. The scores of each subject in each of the EFA-derived empirical factors were included as continuous variables in subsequent analyses. In particular, receiver operating characteristic (ROC) curve analysis and associated statistics—i.e., area under the curve (AUC) and its 95% confidence interval (CI)—were used to evaluate the power of empirical factors in discriminating clusters. All analyses were performed separately for the meninges and WM using the Stata software (version 13.0). The significance level was corrected through the Bonferroni method in order to account for multiple testing. For the correction, the number of EFA-derived empirical factors in the meninges and WM (see the “Results” section) was considered as the number of independent statistical comparisons involving the studied genes. This led to corrected *p* value thresholds of 0.0125 (i.e., 0.05/4) and 0.01 (i.e., 0.05/5) for the comparisons within the meninges and WM, respectively; for the cross-compartment comparisons (i.e., between meninges and WM), the most stringent of the two *p* value thresholds (i.e., 0.01) was used. The robustness of the results was checked by comparing the significance of the observed differences and associations before and after Bonferroni correction.

## Results

### Sample selection and characterization of brain immune infiltrates used for laser capture microdissection

In preliminary experiments, 69 snap-frozen brain tissue blocks from 22 cases with progressive MS, mainly secondary progressive MS, were used to evaluate RNA quality (Additional file 1). These cases were selected because some of the brain tissue blocks analyzed in previous studies were highly inflamed and comprised B cell follicle-like structures in the brain meninges and active WM lesions, which are rarely found in chronic MS stages ([6, 7, 46, 52, 53]; our unpublished observations). No significant correlation was found between postmortem delay and RIN values (Spearman’s correlation coefficient = − 0.278; *p* = 0.123). Brain tissue blocks with RIN values ≥ 6 (*n* = 56 from 20 cases) were analyzed by immunohistochemistry to assess WM inflammatory activity and demyelination; number, size, and cellular composition of WM perivascular immune infiltrates; meningeal integrity; and presence and lymphoid-like organization of meningeal infiltrates. Only tissue blocks with RIN values ≥ 6 and prominent immune infiltration in the WM and/or the meninges were selected for LCM and subsequent RNA analysis (Additional file 1). The final sample cohort consisted of 23 brain tissue blocks from 11 MS cases with a postmortem delay ranging between 7 and 26 h (median value = 15 h). One to three brain tissue blocks per MS case were used for LCM. The demographic and clinical data, postmortem delay, and RIN of brain tissue blocks of the 11 MS cases included in the study are summarized in Table 1.

**Table 1:** Summary of demographic and clinical data of MS cases, brain sample characteristics, and type and number of laser-cut immune infiltrates analyzed in this study

MS case/no. of brain tissue blocks analyzed	Sex/age at death	Disease duration (years)	Cause of death	Postmortem delay (hours)	RNA integrity number	Laser-cut samples used for gene expression analysis		
						No. of meningeal infiltrates	No. of WM perivascular infiltrates	No. of total infiltrates
MS79/3	F/49	21	Bronchopneumonia, MS	7	6–7	3	3	6
MS92/3	F/37	17	MS	26	6.1–7.8	5	8	13
MS121/2	F/49	14	MS	24	6.8, 7.0	n.a.	7	7
MS154/2	F/34	11	Pneumonia	12	6.4, 8.3	2	3	5
MS160/2	F/44	15	Aspiration pneumonia, MS	18	6.0, 6.4	2	n.a.	2
MS176/1	M/37	27	Intestinal obstruction, MS	12	6.7	1	4	5
MS180/3	F/44	18	MS	9	6.4–6.7	7	9	16
MS234/3	F/39	15	Pulmonary embolism, pneumonia	15	7.4–7.9	6	n.a.	6
MS330/1	F/59	40	Pneumonia, MS	21	7.8	2	n.a.	2
MS402/1	M/46	20	Bronchopneumonia, MS	12	7.2	1	1	2
MS407/3	F/44	19	Septicaemia, pneumonia	22	6.2–6.7	7	4	11
No. of laser-cut samples analyzed						36	39	75

All brain tissues analyzed were from persons who died during the progressive phase of MS. The samples used for gene expression analysis include 36 samples from the meninges of 10 MS cases, of which 25 were B cell follicle-like structures and 11 were diffuse infiltrates; 39 samples from the white matter of 8 MS cases, of which 13 perivascular infiltrates were isolated from active lesions, 8 from chronic active lesions, 3 from areas of rarefied myelin with activated microglia, and 15 from non-demyelinated areas with activated microglia

*n.a.* not available

**Fig. 1:**
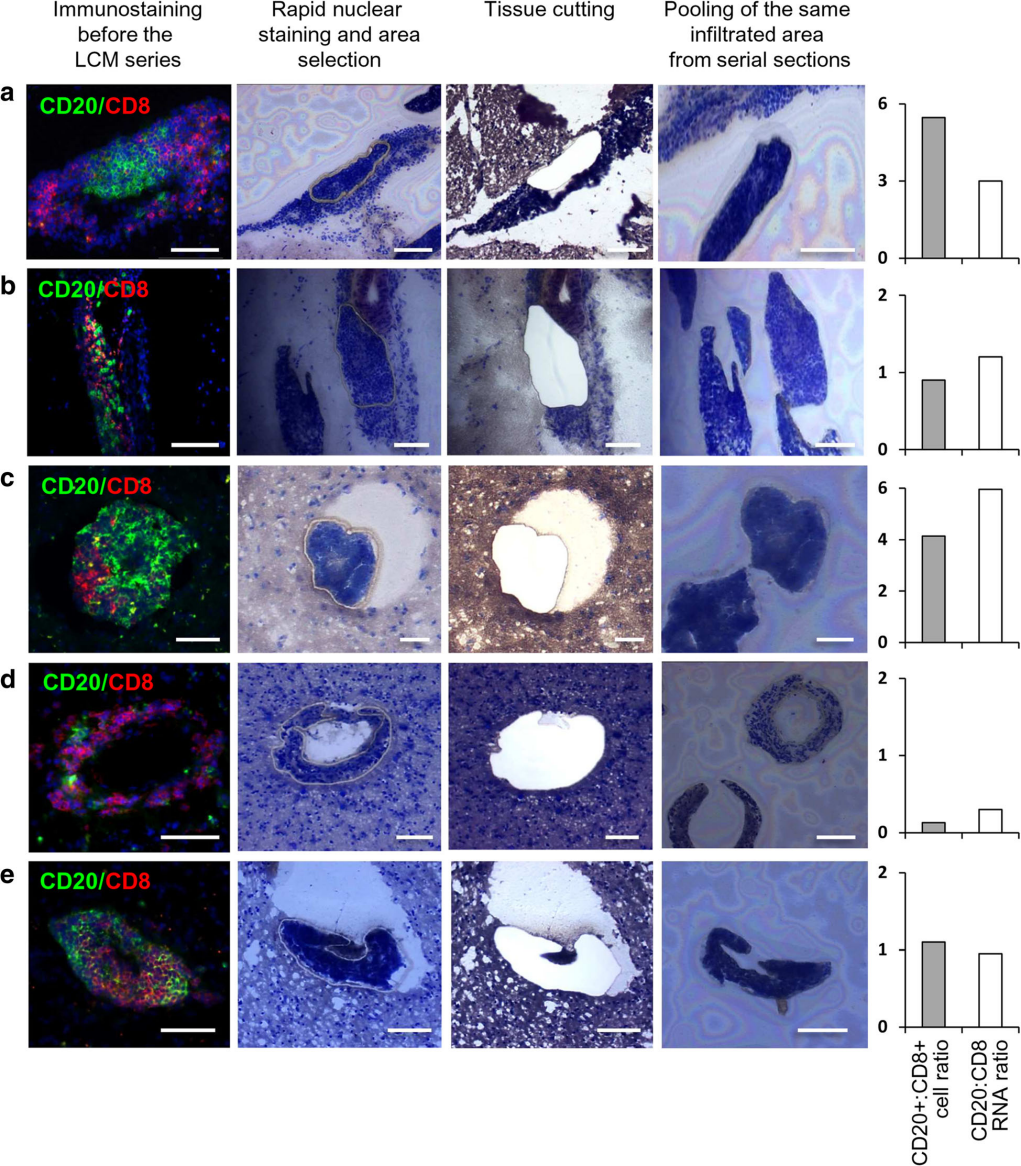
Immunostaining, microdissection, and cell type-specific gene expression of MS brain immune infiltrates. Brain sections were double stained with anti-CD20 and anti-CD8 antibodies to evaluate B cell and CD8+ T cell frequencies and distribution in meningeal immune infiltrates (**a** B cell follicle-like structure; **b** diffuse infiltrate) and WM perivascular immune infiltrates (**c**–**e**). Following rapid nuclear staining, individual immune infiltrates identified in subsequent consecutive brain sections were cut and catapulted from the surrounding tissue into an adhesive cap for RNA extraction. After reverse transcription, cDNA was preamplified for the indicated transcripts and analyzed by real-time PCR. For each microdissected immune infiltrate, the CD20+:CD8+ cell ratio and the CD20:CD8 RNA signal ratio are shown in the bar charts on the right. Bars = 100 μm

Prior to LCM, brain sections were double stained with anti-CD20 and anti-CD8 antibodies to precisely localize the immune infiltrates of interest and evaluate the relative enrichment in B cells and cytotoxic T cells (Fig. 1). These immunostainings were also performed after the series of consecutive brain sections used for LCM to check for the preservation and cellular composition of the isolated infiltrates. It is important to point out that LCM was used selectively in that only large immune infiltrates (as defined in the Methods section) were collected to obtain a sufficient amount of RNA for the subsequent gene expression analysis. Because tissue manipulations for the LCM procedure compromise RNA integrity, we avoided immunostaining of the sections used for LCM and performed rapid fixation and nuclear staining in RNAse-free conditions. In the effort to preserve RNA quality, we minimized LCM duration allowing a range from 2 to a maximum of 7 h to complete the whole procedure (from section cutting to incubation in lysis buffer), depending on the number of inflammatory infiltrates identified in each series of brain sections.

Overall, 87 immune infiltrates (39 from the WM and 48 from the meninges) were collected from the final tissue sample cohort. RNA quantification of the laser cut samples was not possible due to very low RNA content. Hence, after determination of the expression level of the house-keeping gene GAPDH, only samples with a GAPDH Ct value below 28 (*n* = 75) from 11 MS cases were used for the study of cellular and viral gene expression (Table 1). The number of immune infiltrates analyzed for each MS case varied between 2 and 16 (median value = 5) depending on the extent of inflammation in the selected tissue blocks (Table 1). Both meningeal and WM perivascular infiltrates were available from 7 MS cases; only meningeal and WM perivascular infiltrates were obtained from 3 cases and 1 case, respectively. The final collection of laser-cut immune infiltrates included 36 meningeal immune infiltrates, of which 25 were lymphoid-like and 11 were diffuse infiltrates, and 39 immune infiltrates from the WM, of which 13 were isolated from active lesions, 8 from chronic active lesions, 3 from areas of rarefied myelin with activated microglia, and 15 from non-demyelinated areas with activated microglia.

### Immune-related gene expression

Real-time RT-PCR incorporating a multiple target gene preamplification step was used to analyze cellular gene expression in the microdissected brain immune infiltrates. This enhanced method enables analysis of a large number of transcripts even with very low amounts of starting RNA while improving sensitivity for detection of low-frequency transcripts [51]. Preliminary experiments in control lymphoid tissue and EBV+ lymphoblastoid cells showed that the amplification step does not introduce any bias in a comparison analysis of a sample that underwent and a sample that did not undergo the preamplification step [51]. Here, we studied 75 immune-related genes (listed in Table 2) that were selected based on current knowledge of cell subsets, molecules, and pathways involved in inflammatory and immune responses in the CNS [5, 17], lymphoid tissue formation [33, 54], EBV recognition [55, 56], and antiviral immunity [13, 56].

**Table 2:** Expression level and prevalence of cellular genes in laser-cut MS brain immune infiltrates

CD4	0.18 ± 0.13 ^a^ 100%	BAFF	0.038 ± 0.042 96%	IRF7	0.08 ± 0.1 96%
CD8A	2.68 ± 3.65 100%	IL2	0.004 ± 0.009 67%	IFNαR1	0.0087 ± 0.0094 72%
CD20	1.64 ± 2.62 100%	IL4	0.0001 ± 0.0004 11%	Usp18	0.005 ± 0.011 68%
CD138	0.11 ± 0.15 93%	IL9	0.000001 ± 0.000006 1.3%	MXA	0.54 ± 0.62 99%
BCMA	0.016 ± 0.021 87%	IL17A	0.000002 ± 0.000015 1.3%	OAS1	0.08 ± 0.1 100%
NKp46	0.007 ± 0.011 65%	IL22	0.00003 ± 0.00023 3%	IRF8	0.63 ± 0.9 99%
CD56	0.07 ± 0.09 96%	LTα	0.03 ± 0.05 75%	MMP9	0.87 ± 1.33 95%
CD68	1.41 ± 1.34 100%	LTβ	0.11 ± 0.17 99%	COX2	0.08 ± 0.12 95%
CD1a	0.0002 ± 0.0009 20%	TNF	0.05 ± 0.16 84%	iNOS	0.001 ± 0.005 20%
BDCA2	0.0008 ± 0.0024 12%	IL1β	0.07 ± 0.17 83%	CCL2	0.06 ± 0.07 100%
TBX21	0.20 ± 0.29 96%	IL6	0.05 ± 0.08 70%	CCL5	5.66 ± 8.60 100%
EOMES	0.17 ± 0.18 100%	IL10	0.07 ± 0.26 91%	CCL19	5.45 ± 8.23 89%
RORC	0.001 ± 0.003 36%	IL15	0.037 ± 0.041 96%	CCL20	0.004 ± 0.011 43%
FoxP3	0.08 ± 0.15 85%	IL18	0.02 ± 0.05 89%	CCL21	0.10 ± 0.15 85%
CD69	0.27 ± 0.44 100%	p19	0.01 ± 0.02 68%	CXCL10	0.29 ± 0.51 89%
CD161	0.21 ± 0.18 99%	p28	0.01 ± 0.03 36%	CXCL12	0.89 ± 1.06 100%
CD160	0.003 ± 0.008 44%	p35	0.10 ± 0.12 91%	CXCL13	0.22 ± 0.44 84%
Perforin	0.15 ± 0.21 99%	p40	0.006 ± 0.014 55%	CCR5	1.08 ± 2.60 100%
Granzyme A	0.32 ± 0.42 100%	EBI3	0.055 ± 0.049 93%	CCR6	0.05 ± 0.3 81%
Granzyme B	0.01 ± 0.03 51%	GM-CSF	0.0003 ± 0.0009 15%	CXCR3	0.04 ± 0.15 85%
MHC class II	2.83 ± 2.29 100%	IFNβ	0.28 ± 1.43 91%	CXCR5	0.37 ± 1.37 97%
CD86	0.019 ± 0.015 96%	IFNγ	0.01 ± 0.02 68%	RIG1	0.93 ± 1.00 100%
BCL6	1.44 ± 1.78 100%	IL28A	0.00003 ± 0.0002 4%	TLR3	0.005 ± 0.007 64%
CD10	0.003 ± 0.006 45%	IL29	Undetectable 0%	TLR9	0.010 ± 0.013 81%
AID	0.004 ± 0.011 26%	IRF3	1.18 ± 1.55 100%	Ki67	0.065 ± 0.073 83%

^a^Gene expression values are presented as 2^−ΔCt^ relative to GAPDH; mean values ± SD are shown. Percentages represent the fraction of laser-cut immune infiltrates with detectable gene expression. Data obtained in 75 immune infiltrates isolated from 23 brain tissue blocks of 11 MS cases are shown

**Fig. 2:**
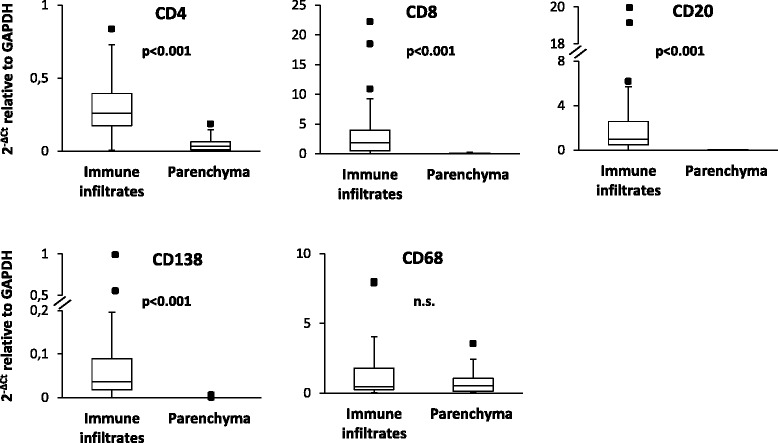
Enrichment in lymphocyte transcripts in immune infiltrates compared to the parenchyma. RNA was extracted and reverse-transcribed from laser-cut meningeal and WM perivascular immune infiltrates and from the adjacent GM and WM parenchyma, respectively. cDNA was preamplified for the indicated transcripts and analyzed by real-time PCR. Gene expression was evaluated in 29 (CD4, CD8, CD20) and 16 (CD138, CD68) matched immune infiltrates and parenchymal samples; significant differences between the two groups were assessed by Mann–Whitney test. Gene expression values are presented as 2^−ΔCt^ relative to GAPDH. The lines inside the boxes represent the median value; boxes extend from the 25th to the 75th percentile, covering the interquartile range (IQR), and whiskers extend from the 25th percentile − 1.5 IQR to the 75th percentile + 1.5 IQR. Maximum outliers outside the whiskers are represented by individual marks. n.s. not significant

To validate the combined LCM/PCR-based approach, in a preliminary analysis, the expression levels of T cell (CD4, CD8), B cell (CD20), plasma cell (CD138), and macrophage (CD68)-specific genes in brain immune infiltrates were compared with those in the adjacent, non-infiltrated parenchyma. The lymphocyte genes were expressed in all (CD4, CD8, CD20) and 90% (CD138) of the immune infiltrates and were undetectable or expressed at a markedly lower level in the parenchyma (Fig. 2). Only CD68 RNA level did not differ significantly between the two sets of samples (Fig. 2), consistently with presence of CD68 immunoreactivity in perivascular/intrameningeal macrophages and intraparenchymal microglia/macrophages [52]. Using CD20 and CD8, we also checked if there was correspondence between RNA signal ratios and immunopositive cell count ratios. It was observed that the CD20:CD8 RNA ratio varied according to the CD20+:CD8+ cell ratio in individual immune infiltrates (Fig. 1). Also, the CD20:CD8 RNA ratio tended to be higher in meningeal infiltrates than in WM infiltrates (*p* = 0.023) and was significantly higher in B cell follicle-like structures than in diffuse meningeal infiltrates (*p* = 0.0001) (data not shown).

Table 2 shows the expression level and prevalence of the 75 selected immune-related genes in the total laser-cut sample cohort. Genes related to the most represented lymphocyte (CD8, CD4, CD20, CD138) and macrophage (CD68) cell populations were expressed at a much higher level and frequency compared to genes related to immune cell subsets, like NK cells (NKp46), conventional DC (CD1a), and pDC (BDCA2), that are known to be minor components of MS brain immune infiltrates [8, 10, 12]. Genes related to T cell growth (IL2) and activation (CD69, CD161); to the differentiation (transcription factors TBX21, encoding Tbet, and EOMES), effector function (the cytolytic enzymes perforin, granzyme A and granzyme B; the cytokines IFNγ, TNF, LTα, and LTβ), and recruitment (the chemokine ligand-receptor pairs CXCL10-CXCR3 and CCL5-CCR5) of type 1 immunity cells; and to regulatory T cells (FoxP3) were expressed much more abundantly and frequently than genes involved in type 2 (IL4) and type 3 immunity (RORC, IL17A, IL22) (as previously reported in ref. [32]) and in the function of other minor T cell subsets (IL9). GM-CSF, a cytokine recently implicated in the pro-inflammatory function of Th1 cells in MS [26], was expressed at a very low level in a minority (15%) of the samples. In line with the ubiquitous presence of macrophages in CNS immune infiltrates, most of the canonical myeloid genes analyzed were found expressed at different levels in 70 to 100% of the immune infiltrates. Included in this group were genes encoding molecules involved in the response to IFNγ (IRF8), antigen presentation (MHC class II), T cell costimulation (CD86), and prostaglandin synthesis (COX-2) and cytokines with B cell growth promoting (BAFF), pro- [IL1β, TNF, IL6, IL12 family subunits (p19, p35, p40), IL15, IL18] or anti-inflammatory [IL10, IL35 (p35/EBI3)] activity. Genes involved in the recognition of EBV DNA (TLR9) and RNA (RIG1, TLR3) and type I IFN pathway (IRF3, IRF7, IFNβ, IFNαR1, MxA, OAS1, Usp18) were found in 70 to 100% of the samples analyzed, while type III IFN genes (IL28A, IL29) were almost undetectable. As to genes involved in immune cell recruitment, the proteolytic enzyme MMP9, which has a key role in cell extravasation [57], and all chemokines (CCL2, CCL5, CCL19, CCL21, CXCL10, CXCL12, CXCL13) and chemokine receptors (CCR5, CCR6, CXCR3, CXCR5) analyzed, except CCL20, were detected in > 80 to 100% of the immune infiltrates. The highly expressed anti-apoptotic gene BCL6 and the relatively less expressed genes CD10 and activation-induced cytidine deaminase (AID) were included in the analysis as germinal center markers [54].

### Differential immune gene expression in immune infiltrates from the meninges and WM

**Fig. 3:**
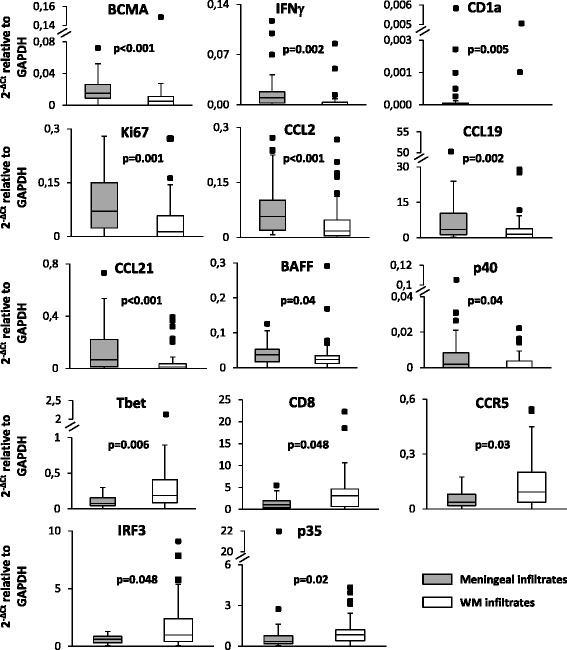
Differential expression of immune-related genes between immune infiltrates isolated from the meninges and the WM. Data obtained in 36 meningeal infiltrates and 39 WM perivascular infiltrates were compared using the Mann–Whitney test; both statistically significant differences (*p* < 0.01, to account for multiple comparisons) and trends (*p* ≥ 0.01, < 0.05) are shown. Gene expression values are presented as 2^−ΔCt^ relative to GAPDH. The lines inside the boxes represent the median value; boxes extend from the 25th to the 75th percentile, covering the interquartile range (IQR), and whiskers extend from 25th percentile − 1.5 IQR to the 75th percentile + 1.5 IQR. Maximum outliers outside the whiskers are represented by individual marks

We next asked whether expression of the immune genes analyzed in this study differed between immune infiltrates isolated from the meninges and from the WM. It was found that genes encoding the BAFF/APRIL receptor BCMA, IFNγ, CD1a, Ki67, CCL2, CCL19, and CCL21 were significantly more expressed in meningeal infiltrates than in WM perivascular infiltrates; also, BAFF and the p40 subunit of IL12/IL23 tended to be more expressed in meningeal infiltrates (Fig. 3). Only TBX21 (encoding Tbet) was significantly more expressed in WM perivascular cuffs than in meningeal infiltrates; two other type-1 immunity-related genes (CD8, CCR5), the IFN-regulatory factor IRF3, and the p35 subunit of IL12/IL35 also tended to be more expressed in WM infiltrates (Fig. 3).

**Fig. 4:**
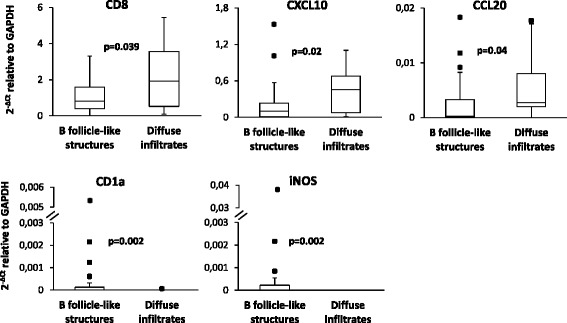
Differential expression of immune-related genes between lymphoid-like and diffuse immune infiltrates isolated from the MS brain meninges. Data obtained in 25 B cell follicle-like structures and 11 meningeal diffuse infiltrates were compared using the Mann–Whitney test; both statistically significant differences (*p* < 0.0125 to account for multiple comparisons) and trends (*p* ≥ 0.0125, < 0.05) are shown. Gene expression values are presented as 2^−ΔCt^ relative to GAPDH. The lines inside the boxes represent the median value; boxes extend from the 25th to the 75th percentile, covering the interquartile range (IQR), and whiskers extend from 25th percentile − 1.5 IQR to the 75th percentile + 1.5 IQR. Maximum outliers outside the whiskers are represented by individual marks

Gene expression was also compared between lymphoid-like and diffuse infiltrates isolated from the meninges. CD8, CXCL10, and CCL20 tended to be more expressed in the diffuse infiltrates, while CD1a and iNOS were significantly more expressed in meningeal B cell follicle-like structures (Fig. 4). This latter result could suggest intrafollicular enrichment of NO-producing myeloid cells, which play a key role in the control of viral infections [58]. None of the genes involved in germinal center function (like BCL6, AID, CD10) were found to be more expressed in meningeal B cell follicle-like structures suggesting that these do not reach the level of functional organization of ectopic B cell follicles with germinal centers present in chronically inflamed tissues of patients with rheumatoid arthritis, myasthenia gravis, and autoimmune thyroiditis [54].

Comparison of gene expression data among infiltrates isolated from different types of WM lesions and non-demyelinated WM areas did not yield significant results.

### EBV gene expression in MS brain immune infiltrates

**Fig. 5:**
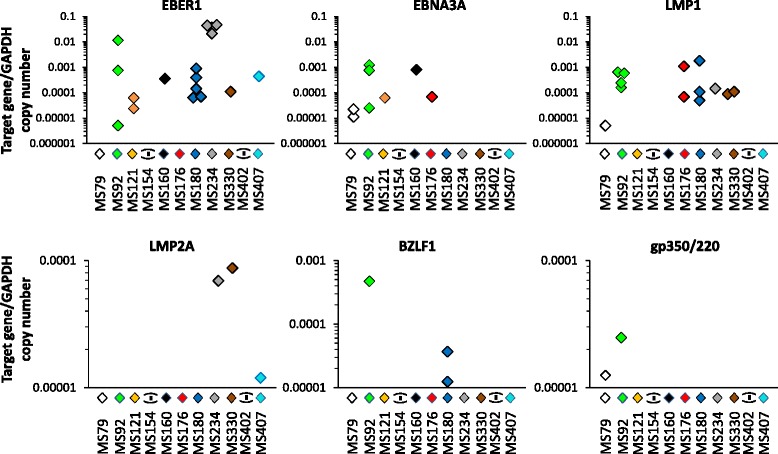
Expression of EBV latent and lytic genes in laser-cut MS brain immune infiltrates. Expression levels of four EBV latent genes (EBER1, EBNA3A, LMP1, LMP2A) and two EBV lytic genes (BZLF1, gp350/220) in immune infiltrates (*n* = 75) isolated from the meninges and WM of 11 MS cases were evaluated using preAmplification ddPCR. EBV genes were detected in samples from 9 MS cases; samples from 2 cases (MS154 and MS402) were negative. The percentages of samples positive for EBV genes were 22.7% (17/75) for EBER1, 10.7% (8/75) for EBNA3A, 17.3% (13/75) for LMP1, 4.0% (3/75) for LMP2A, 4.0% (3/75) for BZLF1, and 2.7% (2/75) for gp350/220. Gene expression values are presented as log of the ratio between target gene and reference gene (GAPDH) copy number

EBV gene expression was investigated in different cDNA aliquots of the samples used for the study of immune genes. Aiming to increase the accuracy in quantifying viral gene expression, we applied PreAmp-ddPCR to study four EBV latent (EBER1, EBNA3A, LMP1, LMP2A) and two EBV lytic (BZLF1, gp350/220) genes. One or more EBV genes were detected in immune infiltrates from 9 of 11 MS cases and in 41.3% of the samples. EBV genes were detected more frequently in meningeal than in WM perivascular infiltrates (55.6 vs 28% of the samples; *p* = 0.006 by Fisher’s exact test), and genes expressed during viral latency were detected more frequently than genes associated with the viral lytic cycle (38.7 vs 6.6% of the samples; *p* = 0.0001 by Fisher’s exact test). EBERs (EBER1/2), the most abundant EBV non-coding small RNA, are expressed in all stages of EBV latency [59, 60]. EBER1 was detected in brain immune infiltrates from 7 of 11 MS cases (Fig. 5). EBNA3A is one of the 10 EBV latent genes expressed in type III latency which is essential for B cell growth/transformation [59, 60]. EBNA3A was detected in samples from 5 of 11 MS cases (Fig. 5). LMP1 and LMP2A are viral genes expressed in type III and type II latency [59, 60] and deliver surrogate B cell survival and differentiation signals (CD40 and B cell receptor, respectively) [59, 60]. LMP1 was detected in 6 of 11 MS cases and LMP2A in 3 of 11 MS cases (Fig. 5). The immediate early gene BZLF1, which encodes a transcription factor involved in the switch from EBV latency to the lytic phase, and gp350/220, which encodes a glycoprotein expressed on the virion envelope [59, 61], were detected in immune infiltrates from two MS cases, respectively (Fig. 5). The EBV genes expressed in individual WM and meningeal immune infiltrates from the 9 EBV+ MS cases are shown in Additional file 7.

### Results of multivariate analysis

The gene expression data obtained in meningeal and WM infiltrates were then analyzed using a multivariate approach. Cluster analysis was used to determine subgroups of MS case-derived samples. Factor analysis was used to group correlated genes into relatively independent factors and verify whether such artificial factors allowed for interpretation of biological processes and associated with sample clusters, neuropathological features, or EBV status.

Factor analysis on gene expression data of meningeal immune infiltrates identified four artificial factors with minimal overlap of genes between factors that accounted for 28.7, 16.8, 10.7, and 6.9% of the variance, respectively (cumulative variance for the four factors is therefore 63.1%). Table 3 shows the genes with the strongest correlation (factor loadings > 0.5) with each factor. Factor 1 comprises genes related to type 1 immunity (CD8, NKp46, EOMES, granzyme A, perforin, IFNγ, LTβ, CCL5), T cell/NK cell activation (CD69, CD161), antigen presentation (MHC class II), and myeloid cell recruitment (CCL2), and response to IFNγ (IRF8). Factor 2 comprises the EBV gene EBNA3A, expressed during latency III or growth program, the master regulator of type I IFN gene expression IRF7, which is induced during EBV latency III [62], and type I IFN-induced genes (MxA, OAS1), chemokine receptors associated with type 1 immunity (CCR5 and CXCR3), and chemokines involved in monocyte (CCL2) and T lymphocyte (CCL19) recruitment. Factor 3 correlates with several genes related to B cell survival and maturation (IL6, IL10, BAFF, BCMA, CD138); the microbial DNA sensor TLR9 that is expressed in B cells, NK cells, myeloid cells, and pDC [56]; and several genes known to be induced upon TLR9 stimulation, like the costimulatory molecule CD86, the cytotoxic T cell/NK cell activating cytokine IL15, MMP9 and the abovementioned IL6 and IL10. Factor 4 associates positively with the viral RNA sensor RIG-I, IRF7 which is activated by RIG-I [62], and the p35 and EBI3 subunits of IL35, an immunosuppressive cytokine produced by regulatory T cells [63], and negatively with CD56. No association was found between factor scores and lymphoid-like or diffuse infiltrates and EBV gene expression in the meninges.

**Table 3:** Factor loadings derived from gene expression data of meningeal immune infiltrates

Gene	Factor 1	Factor 2	Factor 3	Factor 4
CD8	0.73			
Nkp46	0.62			
EOMES	0.85			
CD69	0.60			
CD161	0.70			
Granzyme A	0.85			
Perforin	0.62			
IFNγ	0.55			
LTβ	0.79			
CD56				− 0.55
CD138			0.77	
BCMA			0.56	
MHCII	0.62			
CD86			0.82	
IL6			0.61	
IL10			0.75	
IL15			0.83	
p35				0.53
EBI3				0.58
MMP9			0.69	
IRF7		0.54		0.62
IRF8	0.74			
MxA		0.87		
OAS1		0.91		
BAFF			0.75	
TLR9			0.70	
RIG1				0.72
CCL2	0.53	0.59		
CCL5	0.89			
CCL19		0.80		
CCR5		0.97		
CXCR3		0.97		
EBNA3A		0.97		

Factor loadings > 0.5 in absolute value are shown

**Fig. 6:**
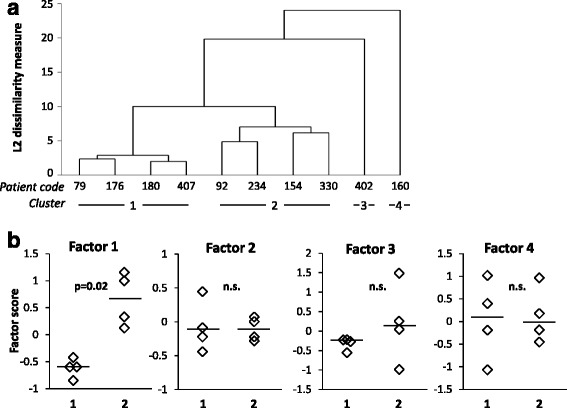
Clustering of MS brain samples and discriminating power of artificial factors derived from gene expression data of meningeal infiltrates. The dendrogram of MS cases based on gene expression data of meningeal infiltrates is shown in **a**. Cluster analysis was carried out on gene expression data of 36 microdissected samples using the average linkage method with the Euclidean distance measure. Panel **b** shows that factor 1, but not factors 2 to 4, discriminates cluster 1 (*n* = 4) and cluster 2 (n = 4). Statistically significant differences were assessed by Mann–Whitney test. Each dot represents the mean factor score value for each MS case; the line marks the median value. n.s. not significant

**Fig. 7:**
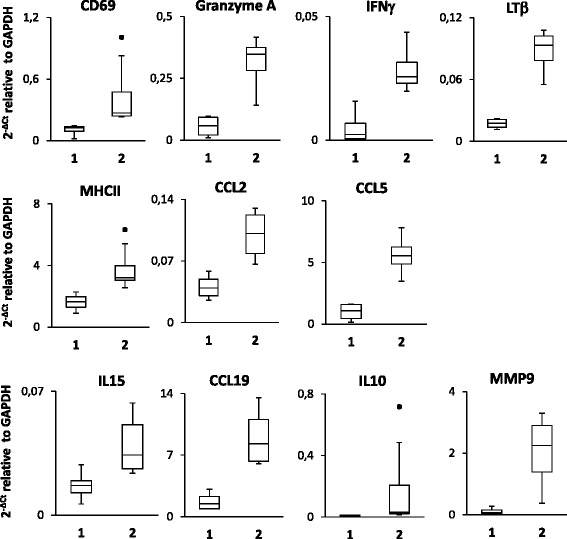
Genes expressed in meningeal infiltrates with discriminatory power in cluster analysis. Expression values of the indicated genes in meningeal immune infiltrates from MS cases grouped into cluster 1 (*n* = 4) and cluster 2 (*n* = 4) are expressed as 2^−ΔCt^ relative to GAPDH. Significant differences between the two groups were assessed by Mann–Whitney test (*p* values = 0.02). The lines inside the boxes represent the median value; boxes extend from the 25th to the 75th percentile, covering the interquartile range (IQR), and whiskers extend from 25th percentile − 1.5 IQR to the 75th percentile + 1.5 IQR. Maximum outliers outside the whiskers are represented by individual marks

Cluster analysis on gene expression data of meningeal infiltrates divided MS patients into four clusters including 4, 4, 1, and 1 case, respectively (dendrogram shown in Fig. 6a). Among the four artificial factors derived from meningeal infiltrate gene expression data, only factor 1 excellently discriminates (AUC = 1.0, 95% CI [1.0, 1.0]) cluster 1 (MS79, MS176, MS180, MS407) from cluster 2 (MS92, MS234, MS154, MS330) MS cases, factor 1 scores being invariably higher in cluster 2 samples (Fig. 6b). The best discriminating genes associated with factor 1 are CD69, granzyme A, IFNγ, LTβ, MHC class II, CCL2, and CCL5 (AUC = 1.0, 95% CI [1.0, 1.0]) (Fig. 7). Cluster 1 and cluster 2 samples are also efficiently discriminated by genes associated with other factors and linked to type 1 immunity activation (IL15), leukocyte extravasation (MMP9), lymphocyte chemoattraction (CCL19), and immunosuppression (IL10) [(AUC = 1.0, 95% CI [1.0, 1.0]) (Fig. 7).

Factor analysis on WM infiltrate gene expression data identified five artificial factors with no gene overlap which explained 24.0, 16.6, 9.4, 7.3, and 6.5% of the variance, respectively (cumulative variance for the five factors is 63.8%) (Table 4). Factor 1 correlates strongly with genes involved in type 1 immunity (IL15, granzyme A, CXCR3), T cell activation (CD161), type I IFN pathway activation (IFNαR1, MxA, OAS1), leukocyte extravasation (MMP9), B cell maturation (BAFF, CD138), and immunoregulation (EBI3 subunit of IL27/IL35). Factor 2 correlates with the regulatory T cell marker FoxP3, the IFN-regulatory factor IRF3, LTβ, the IFNγ-inducing cytokine IL18, and the chemokines CCL2, CCL19, and CXCL10. Factor 3 correlates positively with B cell-related molecules (CD20, the B cell chemoattractant CXCL13 and its receptor CXCR5), TLR9, IRF7, and IRF8 and negatively with CD56. Factor 4 associates with CD8, the viral RNA sensor RIG1, and the proinflammatory cytokines IL1β and TNF. Factor 5 associates with type-1 immunity-related genes, like the transcription factors Tbet and EOMES, and CCL5. Factor scores did not associate with any of the WM areas from which the perivascular cuffs were microdissected nor with EBV gene expression. By applying cluster analysis to WM infiltrate gene expression data, all MS cases, except one (MS121), clustered into a single group indicating no major differences in gene expression (data not shown).

**Table 4:** Factor loadings on gene expression data of WM perivascular immune infiltrates

Gene	Factor 1	Factor 2	Factor 3	Factor 4	Factor 5
CD8				0.72	
TBX21					0.71
EOMES					0.74
Granzyme A	0.64				
LTβ		0.85			
CD161	0.55				
CD56			− 0.59		
FoxP3		0.86			
CD20			0.87		
CD138	0.55				
CD86			0.63		
TNF				0.94	
IL15	0.89				
IL18		0.90			
EBI3	0.62				
IL1β				0.76	
MMP9	0.55				
IRF3		0.81			
IRF7			0.55		
IRF8			0.63		
MxA	0.69				
OAS1	0.62				
BAFF	0.85				
IFNαR1	0.84				
TLR9			0.8		
RIG1				0.86	
CXCL10		0.64			
CCL2		0.83			
CCL5					0.74
CCL19		0.91			
CXCL13			0.75		
CXCR5			0.62		
CXCR3	0.58				

Factor loadings > 0.5 in absolute value  are shown

## Discussion

Aiming at verifying the hypothesis of an association between immune activation and deregulated EBV infection in the MS brain, we examined expression of cellular and viral genes in meningeal and WM immune infiltrates harvested from postmortem brain samples of patients with progressive MS. The application of rigorous criteria for sample selection and the use of enhanced PCR-based methods made it feasible to perform an accurate semi-quantitative gene expression analysis in the laser-cut samples.

The majority (> 80%) of the 75 immune-related genes analyzed were detected at different levels in most or all of the collected brain immune infiltrates. These include genes related to T cell activation, B cell growth and differentiation, pathogen recognition, myeloid cell function, type I IFN pathway activation, and leukocyte recruitment. Among the genes expressed at very low level and/or frequency were genes related to specific T cell subsets and innate immunity cells (DC, pDC), germinal center function, type III IFN, and inflammation (GM-CSF, iNOS). Analysis of the whole laser-cut sample cohort showed that genes involved in type 1 immunity activation and effector functions predominate in brain immune infiltrates as compared to genes involved in type 2 and type 3 immunity. Type 1 immunity relates to a milieu skewed towards cytotoxic functions including enhanced Th1, CD8+ T cell, and NK cell activities [13]. The major function of type 1 immunity is to kill cancer cells and protect against intracellular microbes, including viruses, through direct lysis of infected cells and macrophage activation. The present data corroborate previous studies in postmortem MS brain samples showing that CD8+ T cells displaying proliferative and cytotoxic activity dominate the T cell infiltrate and that IFNγ is a major cytokine produced in CNS immune infiltrates [8, 16, 18–22].

This study also reveals differences in gene expression between WM and meningeal immune infiltrates. Of particular interest is the finding that IFNγ gene expression is significantly higher in meningeal immune infiltrates, despite other genes linked to cytotoxic type 1 immunity (TBX21 encoding Tbet and suggestively CD8 and CCR5) are more expressed in WM perivascular infiltrates. Because IFNγ has a key role in antiviral defense, the more pronounced induction of this cytokine in the meninges could result from wider propagation of EBV infection, as EBV RNA+ samples were two times more frequent in meningeal than in WM infiltrates. Besides IFNγ, the other genes found to be more expressed in the meninges are related to cell proliferation (Ki67), B cell differentiation (BCMA and suggestively BAFF), lipid antigen presentation (CD1a), monocyte (CCL2) and T lymphocyte (CCL19, CCL21) recruitment, and myeloid cell activation (p40 subunit of IL12/23). The finding that the CD1a+ DC subset in humans produces significant amounts of IL12 and displays type 1 polarizing activity [64] could explain stronger type-1 immunity activation in the meningeal compartment. Interestingly, studies performed in mouse models of viral infection in the CNS have shown that CCL19 and CCL21 produced in the meninges are crucial to support recruitment and local reactivation of antiviral CD8+ T cells [65] and that the meninges are a preferential site of accumulation and activity of virus-specific tissue-resident CD8+ memory T cells [66]. Tissue-resident memory T cells are a recently identified subset of memory T cells that persists at sites of previous or ongoing infection where it serves as a self-replenishing pool of memory T cells but also recruits circulating immune cells and plays a key role in antiviral immunity through perforin- and IFNγ-dependent effector mechanisms [67]. The presence of brain-resident memory T cells and their relationship to EBV infection in MS remains to be determined.

Multivariate analysis of gene expression data obtained in MS brain immune infiltrates yielded results that corroborate activation of antiviral immunity in the meninges and WM. Factor analysis on meningeal immune infiltrate-derived data resulted in four factors that potentially mirror the following processes: recruitment and activation of type-1 immunity-related cells displaying cytotoxic function (CCL5, CD8, NKp46, Eomes, CD69, CD161, granzyme A, perforin, IFNγ, LTβ) in conjunction with recruitment and IFNγ-mediated activation of myeloid cells (CCL2, IRF8, MHC class II) (factor 1); type I IFN pathway activation (IRF7, OAS1, MxA) by EBV latency disruption (EBNA3A) in conjunction with leukocyte recruitment (CCL2, CCL19, CCR5, CXCR3) (factor 2); B cell growth and differentiation (IL6, IL10, BAFF, BCMA, CD138) in conjunction with TLR9-mediated induction of leukocyte extravasation (MMP9), T cell costimulatory (CD86), and cytotoxicity-promoting (IL15) activities (factor 3); and type I IFN induction (IRF7) by sensing of viral RNA (RIG-I) and immunoregulation (IL35) (factor 4). Among the factors extracted from WM infiltrate gene expression data, factor 1 may reflect concomitant activation of type I IFN pathway (IFNαR1, MxA, OAS1) and cytotoxic type 1 immunity (CXCR3, IL15, CD161, granzyme A), leukocyte extravasation (MMP9), and B cell differentiation (BAFF, CD138). It is important to recall here that type I IFNs, besides having a direct antiviral action, also promote T cell and NK cell cytotoxicity [68]. Factor 3 links B cell recruitment (CD20, CXCR5, CXCL13) to viral DNA recognition (TLR9) and induction of type I IFN (IRF7), myeloid cell response to IFNγ (IRF8), and T cell costimulation (CD86). Factor 4 links viral RNA sensing (RIG-I) to myeloid cell activation (IL1β, TNF), while factor 5 appears related to type 1 immunity activation (TBX21, EOMES, CCL5). Of interest, among the genes related to type 1 immunity, TBX21, granzyme A, perforin, IFNγ, and CCL5 were found associated with CD8+ T cell activation and/or expansion in the peripheral blood during acute EBV infection [69].

Using cluster analysis, it was possible to subgroup MS case-derived brain samples based on meningeal but not WM perivascular infiltrate gene expression data, a finding that corroborates differences in immune activation between the two compartments. It was found that factor 1 derived from meningeal data and many of the genes correlated with this factor (CD69, granzyme A, IFNγ, LTβ, MHC class II, CCL2, and CCL5) efficiently discriminate two clusters of samples, each comprising four MS cases. These two clusters were also efficiently discriminated by genes associated with other factors, like IL15, MMP9, CCL19, and IL10. These patterns of gene expression most likely capture differences in the strength of the immune response in the meningeal compartment at end-stage disease and are tentatively interpreted as a more prominent attraction of effector cells, antigen presentation, and cytotoxic activity but also induction of negative regulatory mechanisms, in the meninges of cluster 2 compared to cluster 1 MS cases.

This study confirms and extends the results of our previous studies combining in situ hybridization, immunohistochemistry, and LCM/RT-PCR techniques to detect EBV infection and showing that the presence of EBV infected B-lineage cells in the MS brain is accompanied by EBV latency disruption and EBV reactivation [15, 46–50]. The present analysis of viral genes in laser-cut brain immune infiltrates differs from the previous ones [46, 49] in that (i) six EBV genes were analyzed at the same time in a larger sample cohort, allowing to get a broader view of EBV infection programs in WM and meningeal immune infiltrates and (ii) ddPCR was used to accurately evaluate viral gene expression. Extensive brain sampling allowed to detect EBV RNA in the majority (9 out of 11) of the MS cases analyzed and, as mentioned above, more frequently in meningeal infiltrates (56%) than in WM perivascular infiltrates (28%). Abnormal latency state/activation in the MS brain is supported by expression of one or more genes associated with EBV latency programs III and/or II (EBNA3A, LMP1, LMP2A) in samples from all nine EBV+ MS cases, while EBV genes associated with immediate early (BZLF1) or late lytic (gp350/220) infection were detected in only one third of the EBV+ MS cases. The diversity of EBV genes that encode proteins expressed across several viral latent and lytic programs suggests broad EBV protein expression and, potentially, viral antigen processing and presentation within the MS brain. A well-established hierarchy exists among CD8+ T cell responses that target EBV antigens. Immediate early (like BZLF1) and early lytic EBV antigens and latency III antigens EBNA3A/3B/3C are immunodominant, whereas EBV latency II antigens (EBNA1/LMP1/LMP2A) are subdominant [70, 71]. Previous data have highlighted a positive association between MS disease activity (clinical and radiological) and frequency of CD8+ T cells specific for EBV lytic antigens (including BZLF1) in the peripheral blood of patients with relapsing remitting MS [49]. Furthermore, selective accumulation of CD8+ T cells specific for EBV antigens, but not cytomegalovirus or MS-associated autoantigens, has been demonstrated in the CSF of patients with clinically isolated syndrome and definite MS [72–74]. Taken together, the immunological findings in MS patients and the data in postmortem MS brain tissue suggest that EBV could be the main antigenic trigger of an immunopathological, CD8+ T cell-mediated response that damages the brain/spinal cord in MS. This model is consistent with the notion that CD8+ T cells are the main drivers of bystander tissue damage in EBV-associated immunopathologic diseases [41].

## Conclusions

Gene expression analysis of immune cells invading the MS brain confirms profound in situ EBV deregulation and highlights orchestration of local antiviral function, lending support to the idea that EBV-induced immunopathology might cause CNS damage in MS. These results should foster research on cell types that are known to play a key role in EBV control, like NK cells [75] and EBV-specific CD8+ T cells [41], as potentially useful predictors of disease evolution and response to therapy. These results also reinforce the rationale for the use of drugs that, by directly targeting the virus and its cellular reservoir, could be more effective in normalizing an altered EBV-host interaction in MS. For example, B cell-depleting therapies could lower EBV load and hence the burden of EBV-induced immunopathology in MS more efficiently than other drugs [76, 77].

## Additional files

Additional file 1: Sample cohort selected for RNA quality control and neuropathological assessment. The table shows the steps of the quality control analysis performed on fresh frozen postmortem MS brain tissue blocks that led to identify a smaller sample cohort suitable for laser capture microdissection and gene expression analysis. (PDF 353 kb)

Additional file 2: Neuropathological characterization of white matter lesions and meningeal immune infiltrates in postmortem MS brain samples. The figure shows representative images of white matter lesions and areas with different degrees of demyelination and inflammation from which perivascular immune infiltrates were microdissected and of ectopic B cell follicles and diffuse immune infiltrates characterized in the meninges. (PDF 843 kb)

Additional file 3: List of Taqman inventoried assays used to study cellular gene expression. The table lists the immune-related cellular genes and the corresponding Taqman inventoried gene expression assays used in this study. (PDF 358 kb)

Additional file 4: List of Taqman self-designed primers and probes used to study EBV gene expression. The table lists the EBV genes, the GenBank nucleotide sequence accession numbers, and the self-designed primers and probes used in this study to analyze EBV gene expression. (PDF 322 kb)

Additional file 5: Assessment of the specificity of the EBV gene expression assays using droplet digital (dd) PCR. The figure shows the results of a representative experiment performed in EBV+ and EBV− cell lines to verify the specificity of the self-designed EBV gene expression assays in a ddPCR setting. (PDF 669 kb)

Additional file 6: Quantification of EBV transcripts in an EBV+ lymphoblastoid cell line by PreAmp droplet digital (dd) PCR compared to real-time PCR. The figure shows the results of an experiment to verify whether EBV gene expression data obtained using ddRT-PCR were comparable with those obtained using real-time RT-PCR. (PDF 345 kb)

Additional file 7: EBV gene expression in laser-cut immune infiltrates from the MS brain. The table shows the EBV latent and lytic transcripts detected in individual immune infiltrates isolated from brain sections of 9 of the 11 MS cases analyzed. (PDF 339 kb)

## Abbreviations

AID: Activation-induced cytidine deaminase
AUC: Area under ROC curve
BAFF: B cell activating factor
BCMA: B cell maturation antigen
BDCA2: Blood dendritic cell antigen 2
CCL: Chemokine (C-C motif) ligand
CCR: C-C chemokine receptor
CI: Confidence interval
CNS: Central nervous system
Cox-2: Cyclooxygenase-2
CSF: Cerebrospinal fluid
Ct: Threshold cycle
CXCL: (C-X-C motif) chemokine ligand
CXCR: Chemokine (C-X-C motif) receptor
DC: Dendritic cells
ddPCR: Droplet digital PCR
EBER1: EBV-encoded small RNA 1
EBI3: Epstein-Barr virus-induced gene 3
EBNA: EBV nuclear antigen
EBV: Epstein-Barr virus
EFA: Exploratory factor analysis
EOMES: Eomesodermin
Foxp3: Forkhead box P3
GAPDH: Glyceraldehyde 3-phosphate dehydrogenase
GM: Gray matter
GM-CSF: Granulocyte-macrophage colony stimulating factor
IFN: Interferon
IFNαR1: IFNα receptor 1
IL: Interleukin
ILCs: Innate lymphoid cells
iNOS: Inducible nitric oxide synthase
IRF: Interferon regulatory factor
LCM: Laser capture microdissection
LMP: EBV latent membrane protein
LT: Lymphotoxin
MHC: Major histocompatibility complex
MMP: Metalloproteinase
MOG: Myelin-oligodendrocyte glycoprotein
MS: Multiple sclerosis
MxA: Myxovirus resistance A gene
NK: Natural killer
NKp46: Natural killer cell p46-related protein
OAS1: 2′-5′oligoadenylate synthetase 1
PCR: Polymerase chain reaction
pDC: Plasmacytoid DC
PreAmp: Preamplification
RIG1: Retinoic acid-inducible gene 1
RIN: RNA integrity number
ROC: Receiver operating characteristic
ROR: Retinoic acid-related orphan receptor
RT-PCR: Reverse-transcription polymerase chain reaction
Tbet: T box expressed in T cells
Tc: T cytotoxic cells
Th: T helper cells
TLR: Toll-like receptor
TNF: Tumor necrosis factor
Usp18: Ubiquitin-specific peptidase 18
WM: White matter
