# Supplementary material for: Transcriptional profile and Epstein-Barr virus infection status of laser-cut immune infiltrates from the brain of patients with progressive multiple sclerosis
Source: J Neuroinflammation. 2018 Jan 16;15:18. doi: 10.1186/s12974-017-1049-5 (PMC5771146; doi:10.1186/s12974-017-1049-5)
Supplement: Supplementary file 1 — Sample cohort selected for RNA quality control and neuropathological assessment. The table shows the steps of the quality control analysis performed on fresh frozen postmortem MS brain tissue blocks that led to identify a smaller sample cohort suitable for laser capture microdissection and gene expression analysis. (PDF 353 kb) [file 12974_2017_1049_MOESM1_ESM.pdf]

Initial sample cohort selected for RNA quality control and neuropathological assessment

MS case	Sex/ Age at death	Disease duration (years)	Cause of death	Post mortem delay (hours)	Number of brain tissue blocks used for RNA extraction	Number of brain tissue blocks with RIN ≥6	Number of brain tissue blocks used for LCM
MS46**	M/40	24	MS	18	1	1	0
MS79	F/49	21	Bronchopneumonia, MS	7	3	3	3
MS92	F/37	17	MS	26	7	7	3
MS121	F/49	14	MS	24	6	5	2
MS153**	F/50	32	MS	12	3	3	0
MS154	F/34	11	Pneumonia	12	4	4	2
MS160	F/44	15	Aspiration pneumonia, MS	18	7	3	2
MS176	M/37	27	Intestinal obstruction, MS	12	1	1	1
MS180	F/44	18	MS	9	8	7	2
MS229**	M/53	16	MS	13	1	1	0
MS234	F/39	15	Pulmonary embolism, pneumonia	15	4	4	3
MS286**	M/45	16	MS	7	1	1	0
MS289**	M/45	18	MS	9	3	3	0
MS317**	F/48	30	Aspiration pneumonia, MS	21	2	2	0
MS325**	M/51	3	Bronchopneumonia	13	2	2	0
MS330	F/59	40	Pneumonia, MS	21	1	1	1
MS342**	F/35	6	MS	9	1	1	0
MS352*	M/43	19	Bronchopneumonia, MS	26	4	0	0
MS356*	F/45	17	MS	10	3	0	0
MS402	M/46	21	Bronchopneumonia, MS	12	3	3	1
MS407	F/44	19	Septicaemia, pneumonia	22	3	3	3
MS422**	M/58	13	Chest infection, MS	25	1	1	0
Total number of tissue blocks					69	56	23

Frozen brain tissue blocks from 22 patients dying during the progressive phase of MS were included in the quality control analysis. Most patients had developed secondary progressive MS, except MS121 and MS234 cases who had developed relapsing progressive MS, and MS176 and MS325 cases who had developed primary progressive MS. Only tissue blocks with RIN ≥6 underwent

neuropathological analysis. Among these, only tissue blocks containing substantial immune infiltrates in the meninges and/or white matter were used for LCM (last column on the right).

* Cases whose brain samples did not pass the RNA quality control step and were excluded from subsequent neuropathological assessment.

** Cases whose brain samples were excluded from the final sample cohort due to absence of substantial immune infiltrates in the meninges and/or white matter.
