# Supplementary material for: Transcriptional profile and Epstein-Barr virus infection status of laser-cut immune infiltrates from the brain of patients with progressive multiple sclerosis
Source: J Neuroinflammation. 2018 Jan 16;15:18. doi: 10.1186/s12974-017-1049-5 (PMC5771146; doi:10.1186/s12974-017-1049-5)
Supplement: Supplementary file 2 — Neuropathological characterization of white matter lesions and meningeal immune infiltrates in postmortem MS brain samples. The figure shows representative images of white matter lesions and areas with different degrees of demyelination and inflammation from which perivascular immune infiltrates were microdissected and of ectopic B cell follicles and diffuse immune infiltrates characterized in the meninges. (PDF 843 kb) [file 12974_2017_1049_MOESM2_ESM.pdf]

Active WM lesion

Chronic active WM lesion

WM with rarefied myelin

Non demyelinated WM

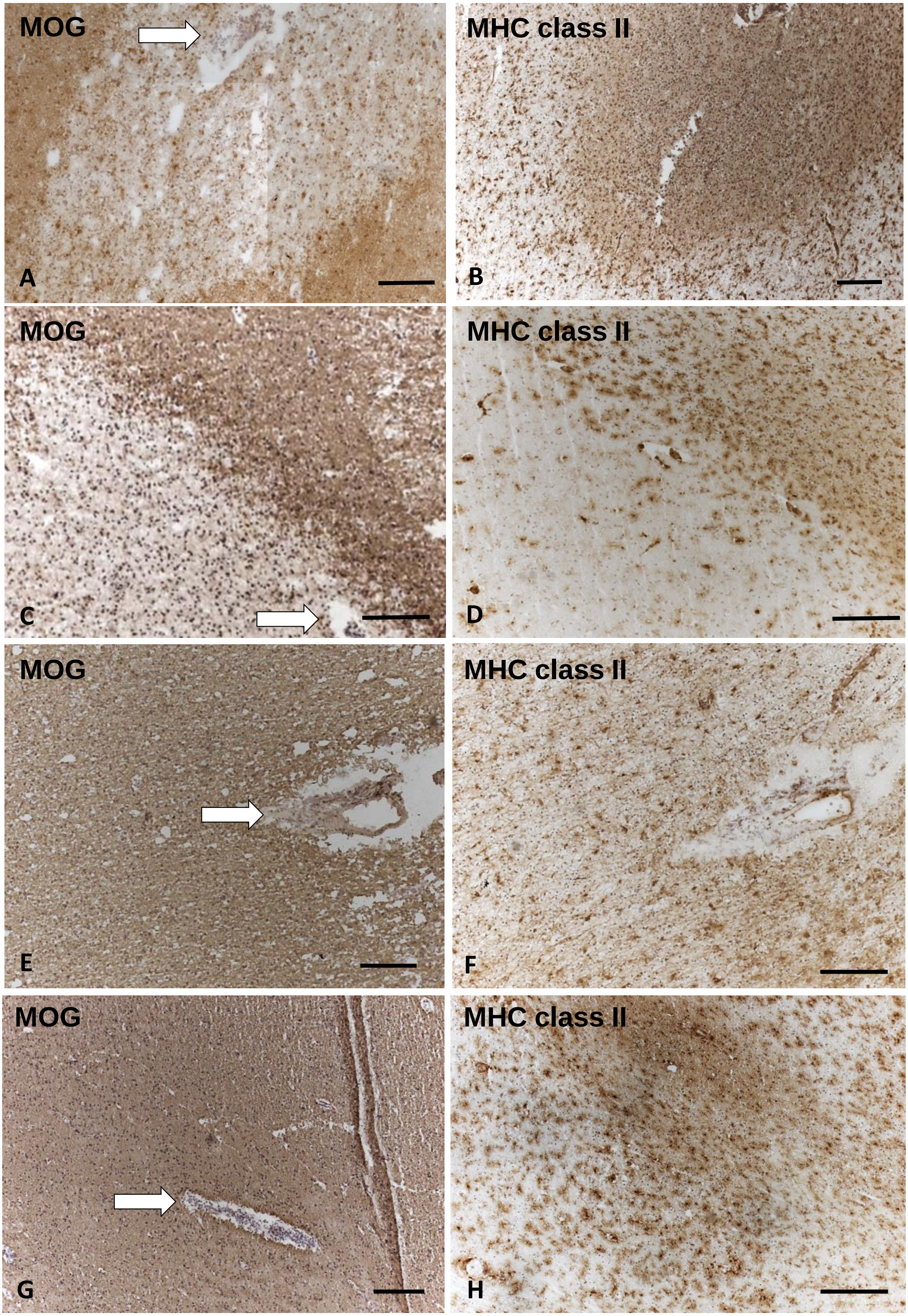

Meningeal infiltrates

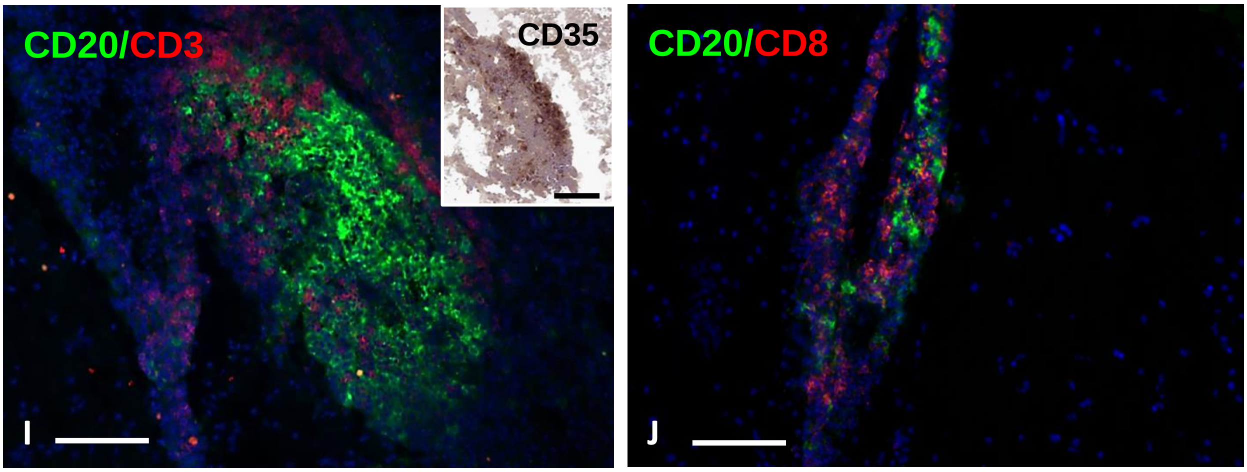

Neuropathological assessment of MS brain samples used for laser capture microdissection. Immunostainings were performed in fresh frozen sections of post-mortem brain tissue from patients with progressive MS to characterize the white matter (WM) regions where the immune infiltrates used for laser capture microdissection and gene expression analysis were localized (A-H), and the lymphoid-like organization or diffuse distribution of microdissected meningeal immune infiltrates (I, J).

WM demyelination and inflammatory activity were assessed in serial brain sections by immunohistochemistry with anti-myelin oligodendrocyte glycoprotein (MOG) (A, C, E, G) and anti-MHC class II antibodies (B, D, F, H), respectively. A, B: active WM lesion with MHC class II+ macrophages and microglia throughout a partially demyelinated area. C, D: chronic active lesion with a central demyelinated area and a hypercellular border containing MHC class II+ macrophages and microglia. E, F: WM area with rarefied myelin and activated microglia. G, H: non demyelinated WM with activated microglia. Arrows in A, C, E, G indicate the perivascular immune infiltrates.

Meningeal immune infiltrates were characterized by indirect double immunofluorescence using anti-CD20 monoclonal antibody and anti-CD3 or anti-CD8 polyclonal antibodies. A meningeal B-cell follicle-like structure is characterized as an aggregate of CD20+ B cells that is partly infiltrated by CD3+ T cells (I) and contains a network of CD35+ stromal cells (inset in I). The diffuse meningeal infiltrate contains intermingled CD20+ B cells and CD8+ T cells (J).

Bars: A, B, E, F, G, H and inset in I = 200 µm; C, D, I, J = 100 µm.
