# Supplementary material for: Transcriptional profile and Epstein-Barr virus infection status of laser-cut immune infiltrates from the brain of patients with progressive multiple sclerosis
Source: J Neuroinflammation. 2018 Jan 16;15:18. doi: 10.1186/s12974-017-1049-5 (PMC5771146; doi:10.1186/s12974-017-1049-5)
Supplement: Supplementary file 3 — List of Taqman inventoried assays used to study cellular gene expression. The table lists the immune-related cellular genes and the corresponding Taqman inventoried gene expression assays used in this study. (PDF 358 kb) [file 12974_2017_1049_MOESM3_ESM.pdf]

List of Taqman inventoried assays used to study cellular gene expression

Gene	Assay code	Gene	Assay code
Glyceraldehyde 3-phosphate dehydrogenase (GAPDH)	Hs99999905_m1	IL22	Hs01574154_m1
CD4	Hs01058407_m1	IL23 subunit alpha (p19)	Hs00900828_g1
CD8A	Hs00233520_m1	Epstein-Barr virus induced gene 3 (EBI3)	Hs01057148_m1
CD20	Hs00544818_m1	IL-27 subunit alpha (p28)	Hs00377366_m1
CD138	Hs00896423_m1	Interferon γ (IFNγ)	Hs00174143_m1
B cell maturation antigen (BCMA)	Hs03045080_m1	Interferon beta (IFN-β)	Hs01077958_s1
Natural Killer Cell p46-related protein (NKp46)	Hs00183118_m1	Interferon regulatory factor 3 (IRF3)	Hs01547283_m1
CD56	Hs00941830_m1	Interferon regulatory factor 7 (IRF7)	Hs00185375_m1
CD68	Hs00154355_m1	Interferon regulatory factor 8 (IRF8)	Hs00175238_m1
CD1a	Hs00381754_g1	Myxovirus (influenza virus) resistance A gene (MxA or Mx1)	Hs00895608_m1
Blood dendritic cell antigen 2 (BDCA2)	Hs01092462_m1	2'-5'oligoadenylate synthetase 1 (OAS1)	Hs00973637_m1
TBX21/T box expressed in T cells (Tbet)	Hs00203436_m1	Ubiquitin specific peptidase 18 (Usp18)	Hs00276441_m1
Eomesodermin (EOMES)	Hs00172872_m1	IFNα receptor 1 (IFNαR1)	Hs01066118_m1
Forkhead box P3 (Foxp3)	Hs01085834_m1	IL28A	Hs00820125_g1
RAR-related orphan receptor C (RORC)	Hs01076122_m1	IL29	Hs00601677_g1
CD69	Hs00934033_m1	Tumor necrosis factor (TNF)	Hs00174128_m1
CD160	Hs00199894_m1	Lymphotoxin (LT) α	Hs04188773_g1
CD161	Hs00174469_m1	LTβ	Hs00242739_m1
Perforin	Hs00169473_m1	Metalloproteinase 9 (MMP9)	Hs00234579_m1
Granzyme A	Hs00989184_m1	Granulocyte-macrophage colony stimulating factor (GM-CSF)	Hs00929873_m1
Granzyme B	Hs01554355_m1	Inducible nitric oxide synthase (iNOS)	Hs01075529_m1
HLA-DRA or major histocompatibility complex II (MHCII)	Hs00219575_m1	Cyclooxygenase-2 (Cox-2)	Hs00153133_m1
CD86	Hs01567026_m1	Chemokine (C-C motif) ligand 2 (CCL2)	Hs00234140_m1
BCL-6	Hs00277037_m1	CCL5	Hs00982282_m1
CD10	Hs00153510_m1	CCL19	Hs00171149_m1
Activation-induced cytidine deaminase (AID)	Hs00757808_m1	CCL20	Hs01011368_m1
B cell activating factor (BAFF)	Hs00198106_m1	CCL21	Hs00989654_g1
Interleukin (IL) 1 β	Hs01555410_m1	C-X-C motif chemokine 10 ligand (CXCL10)	Hs00171042_m1
IL2	Hs00174114_m1	CXCL12	Hs00171022_m1
IL4	Hs00174122_m1	CXCL13	Hs00757930_m1
IL6	Hs00985639_m1	C-C chemokine receptor type 5 (CCR5)	Hs99999149_s1
IL9	Hs00914237_m1	CCR6	Hs01890706_s1
IL10	Hs00961622_m1	Chemokine (C-X-C motif) receptor 3 (CXCR3)	Hs01847760_s1
IL12 subunit beta (p40)	Hs01011518_m1	CXCR5	Hs00540548_s1
IL12 subunit alpha (p35)	Hs01073447_m1	Retinoic acid-inducible gene 1 (RIG1)	Hs00184937_m1
IL15	Hs01003716_m1	Toll-like receptor 3 (TLR3)	Hs01551078_m1
IL17A	Hs00174383_m1	TLR9	Hs00152973_m1
IL18	Hs01038788_m1	Ki67	Hs01032443_m1
