# Supplementary material for: Transcriptional profile and Epstein-Barr virus infection status of laser-cut immune infiltrates from the brain of patients with progressive multiple sclerosis
Source: J Neuroinflammation. 2018 Jan 16;15:18. doi: 10.1186/s12974-017-1049-5 (PMC5771146; doi:10.1186/s12974-017-1049-5)
Supplement: Supplementary file 4 — List of Taqman self-designed primers and probes used to study EBV gene expression. The table lists the EBV genes, the GenBank nucleotide sequence accession numbers, and the self-designed primers and probes used in this study to analyze EBV gene expression. (PDF 322 kb) [file 12974_2017_1049_MOESM4_ESM.pdf]

List of Taqman self-designed primers and probes used to study EBV gene expression

Gene	Forward primer	Reverse primer	FAM-labelled probe
EBV encoded small RNA (EBER) 1	GTTGCCCTAGTGGTTTCG	CCCCGGGACTTGACC	ACACACCGCCAACGCTCAGT
EBV nuclear antigen (EBNA) 3A	GCCCCGTGTCCGGTAG	GAGTTGATCCCCTGGAGATACAG	CACAGGCCCCACCTAC
EBV latent membrane protein (LMP) 1	GGACAACGACACAGTGATGAACA	CATCGGTAGCTTGTTGAGGGT	CCACCACGATGACTCC
LMP2A	GAGATGGCGCCGTTTGAC	TGAGGACAAGTACACATGCCAAAA	ATGCCGCCACAAACA
BZLF1	CTCAACCTGGAGACAATTCTACTGT	TGCTAGCTGTTGTCCTTGGTTAG	CTGCTGCTGCTGTTTG
gp350/220	AGA ATC TGG GCT GGG ACG TT	ACATGGAGCCCGGACAAGT	AGCCCACCACAGATTACGGCGT
