# Supplementary material for: Transcriptional profile and Epstein-Barr virus infection status of laser-cut immune infiltrates from the brain of patients with progressive multiple sclerosis
Source: J Neuroinflammation. 2018 Jan 16;15:18. doi: 10.1186/s12974-017-1049-5 (PMC5771146; doi:10.1186/s12974-017-1049-5)
Supplement: Supplementary file 5 — Assessment of the specificity of the EBV gene expression assays using droplet digital (dd) PCR. The figure shows the results of a representative experiment performed in EBV+ and EBV− cell lines to verify the specificity of the self-designed EBV gene expression assays in a ddPCR setting. (PDF 669 kb) [file 12974_2017_1049_MOESM5_ESM.pdf]

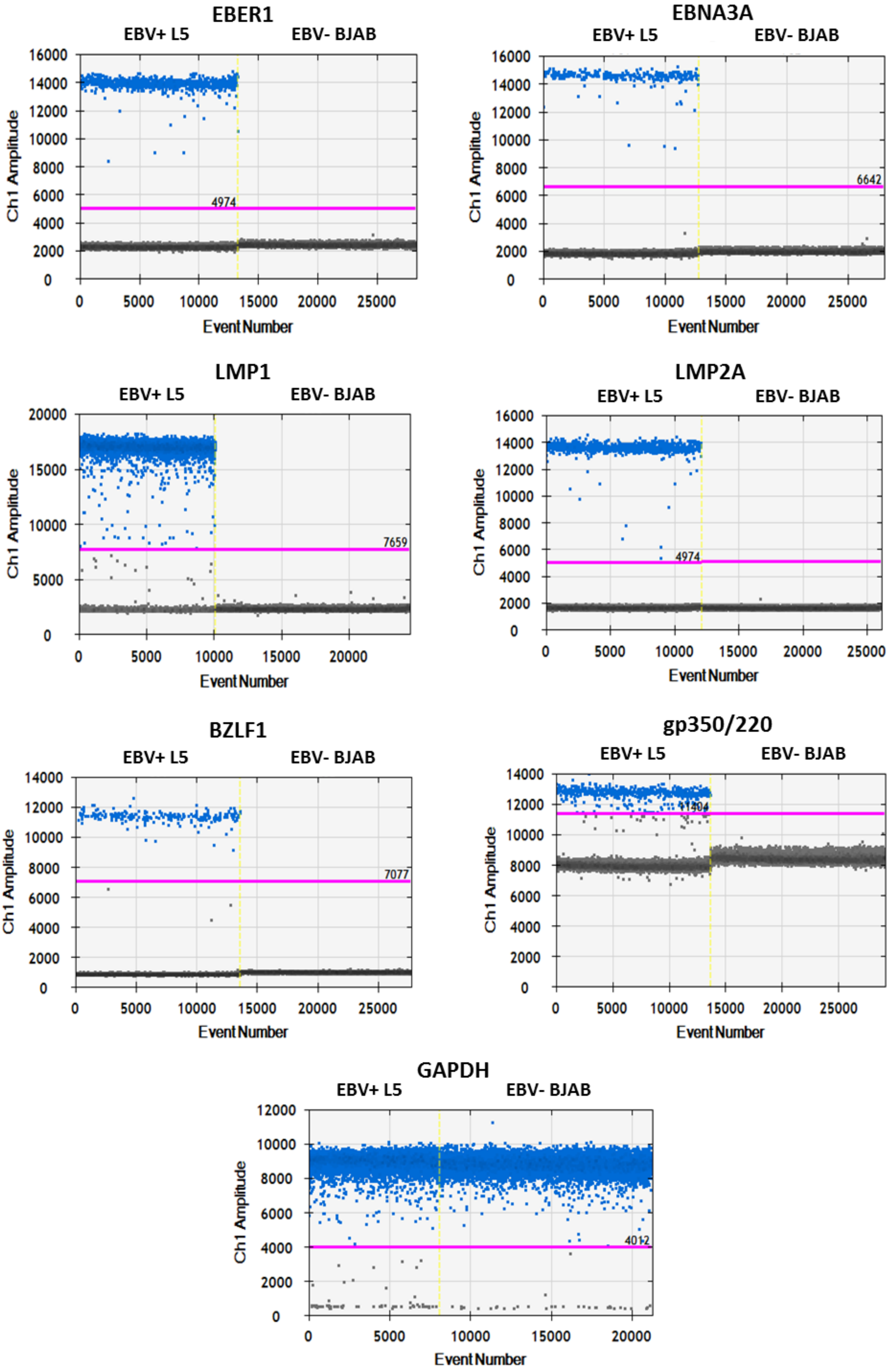

EBER1
EBV+ L5
EBV- BJAB
Ch1 Amplitude
Event Number
4974
EBNA3A
EBV+ L5
EBV- BJAB
Ch1 Amplitude
Event Number
6642
LMP1
EBV+ L5
EBV- BJAB
Ch1 Amplitude
Event Number
7659
LMP2A
EBV+ L5
EBV- BJAB
Ch1 Amplitude
Event Number
4974
BZLF1
EBV+ L5
EBV- BJAB
Ch1 Amplitude
Event Number
7077
gp350/220
EBV+ L5
EBV- BJAB
Ch1 Amplitude
Event Number
11404
GAPDH
EBV+ L5
EBV- BJAB
Ch1 Amplitude
Event Number
4012

Assessment of the specificity of the EBV gene expression assays using droplet digital (dd) PCR. To verify that the specificity of the self-designed EBV gene expression assays used for real time RT-PCR was preserved in a digital PCR system, cDNA from the EBV+ lymphoblastoid cell line L5 and the EBV-negative B lymphoma cell line BJAB were preamplified (14 cycles) for the indicated EBV transcripts together with the housekeeping gene GAPDH and then analyzed using the Bio-Rad QX200 ddPCR System. The blue dots over the threshold (pink line) represent the droplets with detectable target amplification while the underlying grey dots represent the negative droplets. All viral transcripts were amplified in the EBV+ LCL while no signal was present in EBV-negative BJAB cells, confirming the high specificity of the assays. The same amount of cDNA input was used for both cell lines, as demonstrated by the comparable levels of GAPDH detected. For each transcript, one experiment representative of 3 performed is shown.
