# Supplementary material for: Transcriptional profile and Epstein-Barr virus infection status of laser-cut immune infiltrates from the brain of patients with progressive multiple sclerosis
Source: J Neuroinflammation. 2018 Jan 16;15:18. doi: 10.1186/s12974-017-1049-5 (PMC5771146; doi:10.1186/s12974-017-1049-5)
Supplement: Supplementary file 6 — Quantification of EBV transcripts in an EBV+ lymphoblastoid cell line by PreAmp droplet digital (dd) PCR compared to real-time PCR. The figure shows the results of an experiment to verify whether EBV gene expression data obtained using ddRT-PCR were comparable with those obtained using real-time RT-PCR. (PDF 345 kb) [file 12974_2017_1049_MOESM6_ESM.pdf]

Quantification of EBV transcripts in an EBV+ lymphoblastoid cell line by PreAmp droplet digital (dd) PCR versus real time PCR

	ddRT-PCR (Target gene copies/ GAPDH copies)	Real time RT-PCR ($2^{-\Delta Ct}$ to GAPDH)
EBER1	0.012947	0.011395
EBNA3A	0.003165	0.002615
LMP1	0.248234	0.256573
LMP2A	0.011291	0.013249
BZLF1	0.002294	0.002505
gp350/220	0.002218	0.001879

To check whether the EBV gene expression data obtained using ddRT-PCR were comparable with those obtained using real time RT-PCR, 100 ng of cDNA from an EBV+_lymphoblastoid cell line (L5; Veroni et al. J Neuroinflammation 2015;12:132. doi: 10.1186/s12974-015-0353-1) were preamplified (14 cycles) for the indicated EBV genes together with the reference gene GAPDH and analyzed by ddPCR and real time PCR using ABI PRISM 7500 and Bio-Rad QX200 System, respectively. The ddPCR data are expressed as the ratio between target gene copy number and GAPDH copy number, while real time PCR data are expressed as $2^{-\Delta Ct}$ normalized to GAPDH. The relative amount of viral RNA detected with the two techniques is similar for each viral transcript analyzed. Because ddPCR enables absolute and reproducible quantitation of nucleic acids, these findings led us to use PreAmp ddRT-PCR to quantify EBV transcripts in laser-cut immune infiltrates from the post-mortem MS brain.
