# Supplementary material for: Transcriptional profile and Epstein-Barr virus infection status of laser-cut immune infiltrates from the brain of patients with progressive multiple sclerosis
Source: J Neuroinflammation. 2018 Jan 16;15:18. doi: 10.1186/s12974-017-1049-5 (PMC5771146; doi:10.1186/s12974-017-1049-5)
Supplement: Supplementary file 7 — EBV gene expression in laser-cut immune infiltrates from the MS brain. The table shows the EBV latent and lytic transcripts detected in individual immune infiltrates isolated from brain sections of 9 of the 11 MS cases analyzed. (PDF 339 kb) [file 12974_2017_1049_MOESM7_ESM.pdf]

EBV gene expression in laser-cut immune infiltrates from the MS brain

MS case	n° laser-cut immune infiltrates EBV positive/ analysed (% positive)	Localization of EBV positive samples	EBV latent transcripts	EBV lytic transcripts
MS79	2/6 (33%)	Meninges #1	EBNA3A	
		Meninges #2	EBNA3A<br>LMP1	gp350/220
MS92	7/13 (54%)	Meninges #1	LMP1	
		Meninges #2	LMP1	
		Meninges #3	EBER1<br>EBNA3A	gp350/220
		WM #4	EBER1<br>EBNA3A<br>LMP1	
		WM #5	LMP1	
		WM #6	EBNA3A	BZLF1
		WM #7	EBER1	
MS121	2/7 (29%)	WM #1	EBER1	
		WM #2	EBER1<br>EBNA3A	
MS160	1/2 (50%)	Meninges #1	EBER1<br>EBNA3A	
MS176	2/5 (40%)	Meninges #1	LMP1	
		WM #2	EBNA3A<br>LMP1	
MS180	9/16 (56%)	Meninges #1	LMP1	
		Meninges #2	EBER1<br>LMP1	
		Meninges #3		BZLF1
		Meninges #4		BZLF1
		Meninges #5	EBER1	
		WM #6	EBER1	
		WM #7	EBER1	
		WM #8	EBER1	
		WM #9	LMP1	
MS234	4/6 (67%)	Meninges #1	EBER1	
		Meninges #2	EBER1	
		Meninges #3	EBER1<br>LMP2A	
		Meninges #4	EBER1<br>LMP1	
MS330	2/2 (100%)	Meninges #1	EBER1<br>LMP1<br>LMP2A	
		Meninges #2	LMP1	
MS407	2/11 (18%)	Meninges #1	EBER1	
		Meninges #2	LMP2A	

The fraction of microdissected samples with detectable EBV gene expression from 9 of 11 MS cases analyzed and the localization of the EBV+ immune infiltrates for each MS case are shown in the 2nd and 3rd column, respectively. The EBV latent and lytic transcripts detected in the individual immune infiltrates are shown in the 4th and 5th column, respectively.
